# Parental support for physical activity and children’s physical activities: a cross-sectional study

**DOI:** 10.1186/s13102-023-00700-9

**Published:** 2023-07-25

**Authors:** Rikuya Hosokawa, Megumi Fujimoto, Toshiki Katsura

**Affiliations:** 1 Department of Human Health Sciences, Graduate School of Medicine, Kyoto University, 53 Kawara-cho Shogo-in Sakyo-ku, Kyoto, 606-8507 Japan; 2 Faculty of Nursing Science, Osaka Seikei University, Osaka, 533-0007 Japan; 3 Faculty of Nursing, Meiji University of Integrative Medicine, Kyoto, 629-0392 Japan

**Keywords:** Parents, Parental support for physical activity, Children, Physical activities, Sports

## Abstract

**Background:**

Physical activity (PA) in school-aged children is imperative for physical and mental development. However, there has been reduced PA among children in recent years owing to environmental changes, resulting in declining physical strength and athletic ability. Although parents’ and children’s PA has been found to be correlated, the specific parental caregiving attitudes associated with children’s actual levels of PA during school-age years remain unknown. Therefore, this study aimed to examine the relationship between parental support for PA and children’s levels of PA.

**Methods:**

In 2017, a self-reported questionnaire survey was administered to 1,515 third-grade students (8–9 years old) and their caregivers in Nagoya, Aichi, a major metropolitan area in Japan. The main items surveyed included the attitudes of children and parents towards PA and the children’s actual PA level. Multiple regression analysis was conducted with parental involvement related to PA as the explanatory variable and children’s PA as the objective variable.

**Results:**

In total, 717 children with valid responses were included in the analysis. The mean age of the children was 9.08 ± 0.33 years; 366 (51.0%) were boys, and 351 (49.0%) were girls. For moderate-to-vigorous and vigorous PA, PA levels tended to be significantly higher in children whose parents provided logistic support such as enrolling their children in a sports club. However, for moderate PA and walking, PA levels tended to be significantly higher in children whose parents used community resources. Modeling and limiting sedentary activities were not associated with children’s PA.

**Conclusions:**

The results of this study suggest that direct parental assistance for PA such as logistic support encourages children to engage in moderate-to-vigorous PA; in addition, parental use of community resources may encourage children to engage in moderate and light PA such as walking. Conversely, indirect and negative involvement such as modeling and limiting sedentary activities were not associated with children’s PA. Therefore, logistic support may need to be strengthened to encourage moderate-to-vigorous and vigorous PA, which is important for children’s health.

**Supplementary Information:**

The online version contains supplementary material available at 10.1186/s13102-023-00700-9.

## Background

Regular physical activity (PA) is essential for health maintenance [1, 2] and is especially vital in early childhood, as it enables cognitive, physical, emotional, social, and motor learning while contributing to the well-being and healthy development of children [3, 4]. In addition, PA has significant benefits for mental health [5–7], for example, reducing symptoms of depression and anxiety. Moreover, recent studies have suggested that PA may improve children’s cognitive function and academic performance [8, 9].

The World Health Organization (WHO) defines PA as “any physical activity produced by skeletal muscles and requiring energy expenditure” [5–7]; therefore, PA refers to all movements, including engagements during leisure time, transportation, and occupation. The WHO recommends that children and adolescents of 5–17 years of age engage in moderate-to-vigorous PA (MVPA) for an average of at least 60 min/d for a healthy and fit life [6]. Unfortunately, global estimates indicate that more than 80% of children do not meet these recommendations [10–15]. Similarly, in Japan, many children do not meet this recommendation [16], with only approximately 20% of Japanese children and adolescents having MVPA levels that meet the WHO recommendation. Existing longitudinal studies show that the overall amounts of PA and MVPA decline rapidly after puberty [17]. Low PA levels and failure to meet PA recommendations are associated with profound consequences for children’s health including increased risk for obesity, low bone density, low physical fitness, and other significant health problems [18].

Furthermore, children who do not engage in PA are denied the positive social and emotional benefits that can be derived from participation such as higher self-esteem, lower anxiety levels, and lessened stress. Moreover, regular PA helps prevent and manage non-communicable diseases such as heart diseases, stroke, diabetes, and some types of cancer. In addition, it can help prevent high blood pressure, maintain a healthy weight, and improve mental health, quality of life, and sense of well-being [10]. Researchers have found that PA behaviors and habits can be tracked from childhood through adulthood [19]; because physically active children are more likely to become active adults, an active lifestyle should be promoted from childhood. Hence, it is important to ensure that young people are engaging in adequate PA levels to support their current and future health status.

Various PA types undertaken during childhood are associated with improved physical and psychological health outcomes throughout life, and developing diverse basic physical abilities during childhood may help establish a lifelong commitment to PA [20]. Basic physical abilities include motor skills (e.g., running and jumping), object control (e.g., throwing and catching), and stability (e.g., balancing and twisting), which are all considered building blocks of movement and foundation for participation in sports and PA [21]. Basic physical abilities in elementary school children are positively correlated with various health benefits such as greater PA participation, increased cardiorespiratory endurance, higher self-esteem, and lower risk for overweight and obesity [22, 23]. Thus, improving basic physical abilities in elementary school children may improve their involvement in PA while enabling and encouraging them to lead active and healthy lifestyles [24].

The decline in Japanese children’s physical fitness and athletic ability is remarkable. The national physical fitness test, which examines various physical fitness elements of the body, shows that the overall scores of female and male children have been on a yearly decline since their peaks in 1978 and 1980, respectively [25]. The reasons for this are considered to include changes in urban structure and a rise in the use of mobile devices such as smartphones. There is an urgent need for maintaining and promoting PA among children for children’s health habits.

Parental relationships typically serve as the primary socializing agent in the lives of young children. Researchers have found a positive correlation between parents and children’s PA, suggesting that children’s exercise habits are influenced by those of their parents [26, 27]. A meta-analysis showed that parental support and modeling behavior are related to children’s PA [28]. In addition, a previous study used an ecological model to identify the variables associated with PA in children, including psychosocial, social, cultural, and environmental factors [29]. Within the social and cultural categories, parental support including encouragement and financial, instrumental, and emotional support for PA were associated with children’s PA [30, 31]. Furthermore, a positive association has been shown between both parental reports and children’s perceptions of parental PA support and children’s PA levels owing to direct strategies (e.g., taking children to places where they can be active) and indirect strategies (e.g., modeling and verbal encouragement) [32, 33]. Parental PA support encompasses instrumental and direct support (e.g., sharing sports equipment, transportation to practices, and engaging in PA together), psychological and emotional support (e.g., personal incentives, motivation, and encouragement to practice), and informational support (e.g., orientation on the importance of PA and appropriate ways to be involved such as counseling and conversation) [34, 35]. This suggests that the role of social support may partially explain the increase in self-efficacy, which in turn may influence the commitment to participate in and maintain PA. Thus, it is theoretically expected that parents who engage in PA would endorse this behavior, as children tend to spend more time with their parents and share a common contextual environment. Indeed, as proposed in the social cognitive theory, children are more likely to adopt parent-like behaviors, implying that parents who engage in PA can provide social support for their children to adopt this behavior. However, many parents lack the knowledge and skills to optimally support PA, and the specific parental caregiving attitudes that are associated with children’s actual PA levels during school-age years are unclear [36–39]. Therefore, this study aimed to examine the relationship between parental support for PA and children’s actual levels of PA. We hypothesized that parents’ characteristic support of PA to their children would be related to their children’s PA level.

## Methods

### Participants

This study was part of a research project which aimed to examine the effects of parenting environment on children’s social development and behavior. We recruited all 5-year-old children from 52 kindergartens and 78 preschools in Nagoya, Aichi, a major metropolitan area in Japan, in 2014. Afterward, we conducted annual surveys of the participants and used follow-up sample data from 2017. At baseline in 2014, 3,314 people participated in the survey; however, owing to relocation and other dropouts, 1,515 people were ultimately included in the 2017 survey. A self-reported questionnaire was given to the parents (n = 1,515) of the children who were 8–9 years old and in the third grade at a follow-up in 2017. This age group was selected as the middle of elementary school is an important period when children begin to develop exercise habits, especially in Japanese elementary schools, where club activities are initiated. A total of 803 questionnaire responses were obtained. Children diagnosed with developmental disabilities (n = 53) and those whose parents did not complete the required questionnaire items (n = 33) were excluded from the analysis. A total of 717 valid responses from children were ultimately included in the study analysis.

### Data collection

Parents completed the Activity Support Scale for Multiple Groups (ACTS-MG) survey (Supplementary Tables 1, Additional File 1), which is a 12-item questionnaire that assesses parental support for exercise and consists of four subscales representing logistic support (e.g., I take my child to places where he/she can be active), modeling (e.g., I encourage my child to be physically active by leading by example), use of community resources (e.g., I encourage my child to use resources in our neighborhood to be active), and limiting sedentary activities (e.g., I limit how long my child plays video games) [40]. Items are scored on a 4-point Likert-type scale ranging from 1 (strongly disagree) to 4 (strongly agree), with higher scores indicating greater parental support for PA. The validity and reliability of this questionnaire have been previously demonstrated [40].

The WHO developed the International Physical Activity Questionnaire (IPAQ) to assess and compare the number of days and duration of high- and moderate-intensity PA per week [41]. The IPAQ is available in two versions: (1) an extended version that assesses PA during daily life situations such as at work, while traveling, at home, and during leisure time, and (2) a short version that focuses only on PA intensity. The reliability and validity of these questionnaires have been previously evaluated [41, 42]. The short form of the IPAQ (IPAQ-SF) was used in this study, in which participants were asked to recall the number of days they performed each activity in the last 7 d (frequency), the time they were involved in each daily activity (duration), and the average time they spent in sedentary behavior. For this survey, participants were asked to indicate the number of days and average duration of high- and moderate-intensity PA and walking in a week. For calculating energy expenditure, the amount of PA per week was estimated and expressed in metabolic equivalent of task (MET)-min/week (MET-min/week). The MET assignments for each PA intensity level were walking (3.3 METs; e.g., walking at school and home, walking to travel from place to place, and any other walking done solely for recreation, sport, exercise, or leisure) and moderate (4 METs; e.g., carrying lightweight loads, bicycling at a regular pace, or playing doubles tennis) and high (8 METs; e.g., heavy lifting, digging, aerobics, or fast bicycling) levels [42].

We collected self-reported demographic information regarding the children’s sex, family structure (single or two parents, with or without siblings), household income, and parental education level. To account for these confounding factors, indicators of sex, family structure, household income, and parental education level were included as covariates.

### Ethics statement

The parents/guardians of the children were informed of the purpose and procedures of the study and told that their participation in the baseline survey was voluntary. Parents provided written informed consent for themselves and on behalf of their children to participate in the study, with the understanding that the study would consist of both baseline and follow-up surveys. Ethical approval for this study was obtained from the Ethics Committee of the Kyoto University Graduate School and Faculty of Medicine (approval number E2322). This study was conducted in accordance with the principles of the Declaration of Helsinki.

### Data analysis

To analyze how parental caregiving attitudes regarding school-age PA are related to children’s PA, multiple regression analysis was performed with parental involvement related to PA (ACTS-MG) as the explanatory variable and children’s PA (IPAQ) as the objective variable. Indicators of sex, family structure, household income, and parental education level were considered adjustment variables. Two models were used for forecasting. In model 1, each predictor variable was entered separately, and univariate associations with each outcome were assessed. In model 2, all predictor variables were entered simultaneously. Multicollinearity was assessed using variance inflation factors (VIFs); VIF values < 2 indicated no multicollinearity among the predictors. Statistical significance was set at p < 0.05. Statistical Package for Social Sciences version 29 (IBM Corp., Armonk, NY, USA) was used for statistical analysis.

## Results

The demographic characteristics of all participants are shown in Table 1. The mean age of the participants was 9.08 ± 0.33 years, with 366 (51.0%) male and 351 (49.0%) female children (Table 1).

**Table 1:** Demographic characteristics (n = 717)

*Description*	*n*	*%*
**Child’s sex**		
Male	366	51.0
Female	351	49.0
**Family composition**		
Single-parent family	42	5.9
Two-parent family	675	94.1
**Siblings**		
No	108	15.1
Yes	609	84.9
**Annual household income (million JPY)**		
< 5	224	31.2
≥ 5–6	225	31.4
≥ 7	256	35.7
**Maternal education level**		
Middle school or high school	146	20.4
Junior college or vocational school	290	40.4
University or graduate school	273	38.1
**Paternal education level**		
Middle school or high school	168	23.4
Junior college or vocational school	101	14.1
University or graduate school	425	59.3

Abbreviations: *n*: number; JPY: Japanese yen

The relationship between the children’s attributes and PA was analyzed (Table 2). The results showed that male children were more physically active than female children in terms of MVPA, vigorous PA, and moderate PA. Children with siblings were more physically active than those without siblings in terms of MVPA and vigorous PA. Children whose fathers had attended middle or high school were more physically active than other children in terms of walking. Overall, the minimum required PA level for adults (23 MET/week) was met by 679 (94.7%) participants.

**Table 2:** Association between children and familial characteristics and physical activities (n = 717)

	Moderate-to-vigorous physical activity			Vigorous physical activity			Moderate physical activity			Walking		
	M	SD	p	M	SD	p	M	SD	p	M	SD	p
Child’s sex												
Male	1399.54	1702.98	< 0.001*	1080.16	1535.66	< 0.001*	319.38	543.11	0.001*	640.68	839.15	0.286
Female	787.07	1061.61		580.74	947.86		206.32	364.69		576.70	760.64	
**Family composition**												
Single-parent family	760.24	1130.67	0.120	497.14	863.53	0.083	263.10	493.51	0.989	849.55	1140.98	0.160
Two-parent family	1120.83	1473.14		856.74	1325.52		264.09	466.25		594.42	774.51	
**Siblings**												
No	749.07	774.48	< 0.001*	530.74	647.24	< 0.001*	218.33	353.16	0.271	528.92	528.56	0.258
Yes	1161.89	1539.19		889.75	1383.20		272.14	484.79		623.63	840.53	
**Annual household income (million JPY)**												
< 5	952.86	1445.29	0.153	682.68	1278.77	0.088	270.18	487.95	0.820	684.05	1025.19	0.161
≥ 5–6	1202.28	1330.75		926.04	1217.29		276.23	475.38		618.27	652.67	
≥ 7	1157.34	1577.05		906.80	1407.45		250.55	449.68		543.28	702.94	
**Maternal education level**												
Middle school or high school	995.96	1361.89	0.396	718.63	1174.98	0.440	277.33	481.71	0.073	632.59	814.97	0.729
Junior college or vocational school	1081.08	1573.22		862.41	1482.14		218.66	383.49		626.18	932.15	
University or graduate school	1191.83	1387.69		883.81	1174.57		308.02	537.88		579.10	639.23	
**Paternal education level**												
Middle school or high school	1068.11	1456.75	0.927	760.12	1268.73	0.622	307.99	551.16	0.304	773.24	983.10	0.002*
Junior college or vocational school	1102.77	1751.69		842.77	1524.68		260.00	499.90		479.81	496.56	
University or graduate school	1120.35	1407.78		877.46	1289.26		242.89	412.73		554.32	730.70	

Abbreviations: M: mean, SD: standard deviation, JPY: Japanese yen. * Indicates statistically significant difference. For calculating energy expenditure, the amount of physical activities and walking per week was estimated and expressed in metabolic equivalent of task (MET)-min/week (MET-min/week), and the MET assignments for each intensity level of physical activities were walking (3.3 METs), moderate (4 METs), and high (8 METs) levels

We further analyzed the relationship between parental caregiving attitudes and children’s PA (Table 3). The results showed that in model 1, in which each explanatory variable was entered separately, the higher the parental support for logistic support, modeling, and use of community resources, the higher the PA level, thus showing statistical significance. In model 2, where all the explanatory variables were entered simultaneously, PA tended to be significantly higher with stronger parental support towards logistic support.

**Table 3:** Association between parental attitudes that support physical activity and children’s physical activities (n = 717)

	Model 1					Model 2				
	B	SE	β	p	Adjusted R^2^	B	SE	β	p	Adjusted R^2^
Moderate-to-vigorous physical activity										
Logistic support	228.450	35.183	0.238	< 0.001*	0.107	219.856	44.851	0.227	< 0.001*	0.116
Modeling	72.836	25.133	0.109	0.004*	0.063	-16.558	30.276	-0.025	0.585	
Use of community resources	142.122	37.652	0.142	< 0.001*	0.070	44.420	46.246	0.045	0.337	
Limiting sedentary activities	45.049	31.507	0.054	0.153	0.055	-7.850	33.153	-0.009	0.813	
**Vigorous physical activity**										
Logistic support	198.840	31.731	0.231	< 0.001*	0.094	197.819	40.474	0.228	< 0.001*	0.103
Modeling	67.384	22.659	0.112	0.003*	0.056	-3.242	27.322	-0.005	0.906	
Use of community resources	107.500	34.079	0.120	0.002*	0.066	10.605	41.733	0.012	0.799	
Limiting sedentary activities	38.431	28.411	0.051	0.177	0.047	-6.817	29.918	-0.009	0.820	
**Moderate physical activity**										
Logistic support	29.610	11.562	0.098	0.011*	0.028	22.037	14.661	0.072	0.133	0.046
Modeling	5.452	8.106	0.026	0.501	0.018	-13.316	9.897	-0.063	0.179	
Use of community resources	34.622	12.139	0.110	0.004*	0.027	33.815	15.117	0.108	0.026*	
Limiting sedentary activities	6.618	10.105	0.025	0.513	0.016	-1.033	10.837	-0.004	0.924	
**Walking**										
Logistic support	29.038	19.652	0.057	0.140	0.011	-0.864	24.898	-0.002	0.972	0.033
Modeling	18.360	13.717	0.052	0.181	0.009	-2.753	16.807	-0.008	0.870	
Use of community resources	61.866	20.528	0.117	0.003*	0.021	68.298	25.673	0.129	0.008*	
Limiting sedentary activities	5.409	17.123	0.012	0.752	0.008	-8.557	18.404	-0.019	0.642	

Note: Model 1: independent variables were entered individually; Model 2: all independent variables and adjusted variables (children’s sex, family composition, family income, and parental educational attainment) were entered simultaneously. Abbreviations: B: unstandardized coefficient, SE: standard error, β: standardized coefficient. * Indicates a statistically significant difference

In model 1, MVPA tended to be significantly higher in cases where parents provided logistic support (β = 0.238, p < 0.001, adjusted R^2^ = 0.107), showed modeling behavior (β = 0.109, p = 0.004, adjusted R^2^ = 0.063), and used community resources (β = 0.142, p < 0.001, adjusted R^2^ = 0.070). In model 2, PA tended to be significantly higher when parents’ attitudes towards logistic support (β = 0.227, p < 0.001) were higher (adjusted R^2^ = 0.116).

In model 1, vigorous PA tended to be significantly higher in cases where parents provided logistic support (β = 0.231, p < 0.001, adjusted R^2^ = 0.094), showed modeling behavior (β = 0.112, p = 0.003, adjusted R^2^ = 0.056), and used community resources (β = 0.120, p = 0.002, adjusted R^2^ = 0.066). In model 2, PA tended to be significantly higher when parents’ attitude towards logistic support (β = 0.228, p < 0.001) was higher (adjusted R^2^ = 0.103).

In model 1, moderate PA tended to be significantly higher in cases where parents provided logistic support (β = 0.098, p = 0.011, adjusted R^2^ = 0.028) and used community resources (β = 0.110, p = 0.004, adjusted R^2^ = 0.027). In model 2, PA tended to be significantly higher when parental use of community resources (β = 0.108, p = 0.026) was higher (adjusted R^2^ = 0.046).

In model 1, walking tended to be significantly higher in cases where parents provided parental support towards the use of community resources (β = 0.117, p = 0.003, adjusted R^2^ = 0.021). In model 2, PA tended to be significantly higher when parental use of community resources (β = 0.129, p = 0.008) was higher (adjusted R^2^ = 0.033).

We further analyzed the results by sex (Supplementary Tables 2 and 3, Additional File 2). Although there were some differences, the results were similar to those obtained during the analysis for boys and girls. However, in a separate supplementary analysis for boys and girls, the “use of community resources” and “limiting sedentary activities” for walking tended to differ. A reason for this is that the average time spent using smartphones and other mobile devices in this study was 36.46 (standard deviation [SD] = 39.50) min for girls and 53.69 (SD = 52.50) min for boys (independent t-test < 0.001). This does not imply that boys are dependent, but instead that they are in a situation where the customary use of smartphones and other mobile devices has become ingrained. Therefore, the relatively less aggressive approaches of “use of community resources” and “limiting sedentary activities” may have effectively increased the exercise opportunities for girls.

## Discussion

This study’s results showed that in MVPA and vigorous PA, PA tended to be significantly higher when the parent’s attitude towards logistic support for PA was more evident. Conversely, for moderate PA and walking, PA tended to be significantly higher with higher parental support towards the use of community resources. Thus, MVPA and vigorous PA were associated with parental logistic support for PA such as specific admission to a sports club, whereas moderate PA and light forms of PA such as walking were associated with parental use of community resources.

The relationship between a child’s attributes and PA was analyzed. We found that male children were more physically active than female children in terms of MVPA, active PA, and moderate PA, which was consistent with the results of previous studies [1, 2]. Considering MVPA and active PA, children with siblings were more engaged in PA, and the presence of siblings at home may increase children’s PA levels. In walking, children whose fathers attended middle and high school were more active in walking than other children. Although no income association was found, it is possible that children whose fathers are the primary source of income may live further away from public transportation such as train stations, and that such differences in living environments may make a difference in the amount of walking.

In this study, the higher the parental logistic support for PA, the higher the children’s MVPA and vigorous PA levels. In addition, previous studies have suggested that parental logistic support is effective in promoting PA, whereas low logistic support has been linked to low PA [43, 44]. In addition to other healthy behaviors, children and adolescents can prevent many chronic diseases by engaging in MVPA [45–47]. Because a substantial number of people do not meet the recommended guidelines for PA [48], logistic support from parents may be an important approach to address this issue in children.

Model 1 showed a positive association between the explanatory variables for the individual nurturing attitudes and PA; however, model 2 showed no significant association between the explanatory variables and PA. As for nurturing attitudes that promote PA, the effect may be relatively low. In developing behavioral patterns, children tend to imitate social behaviors that exemplify the daily behavioral routines of their social models, parents, siblings, and other significant persons. Therefore, children learn by observing and imitating others, including PA habits [49]. In other words, parents should not only convey the importance of behavior but should also demonstrate active participation [50]. Regarding the results for model 1, it may be effective to some extent for parents to encourage their children to be physically active by setting an example, as well as showing their children how they personally enjoy exercise and PA. However, the results of the present study suggest that specific logistic support for PA may be more important than modeling in school-age children. Two types of parenting attitudes promote PA: direct strategies (e.g., taking children to places where they can be active) and indirect strategies (e.g., modeling and verbal encouragement); however, direct strategies seem to be more effective. The reason the association between PA and modeling was lower than that between PA and logistic support may be associated with the amount of time parents and children spend together. While children spend more time with their parents in early childhood, as they reach school age, the target of modeling moves to other adults, such as teachers and peers, instead of parents. Therefore, modeling may not have been noticeable in the present study.

Furthermore, our results showed that higher use of community resources was associated with higher levels of moderate PA and walking. Understanding the social and environmental factors influencing PA is crucial to effectively supporting children to increase their PA behaviors. MVPA is essential, but moderate and walking PA is also important for children’s health. Playing and walking provide some PA and are associated with other behaviors that further increase PA levels [51, 52]. In previous studies, high levels of PA and walking outside of school appeared to be significantly associated with areas of high urban density and mixed-use facilities, especially among older children and adolescents [53]. In addition, proximity to recreational facilities appears to predict PA levels among youth. For younger children, environmental influences affect their parents’ decisions to dictate their children’s behavior. For older children and youth to benefit from independent mobility in terms of health and development, reducing exposure to traffic, increasing on-street supervision through neighborhood and building design, encouraging walking locally, and prioritizing walking and bicycle use is important. In a previous study, children’s participation in PA was positively correlated with publicly provided recreational infrastructure (access to recreational facilities and schools) and transportation infrastructure (presence of sidewalks and controlled crossings, access to destinations, and public transportation) [54]. However, transportation infrastructure (number of roads crossed, traffic density, and speed) and community conditions (crime and community poverty) were negatively correlated with children’s participation in PA [54]. Publicly provided recreational infrastructure (access to recreational facilities and schools) and transportation infrastructure may increase daily PA. From the present results, the use of community resources was positively associated with moderate PA and walking.

Restricting access to sedentary activities was not significantly associated with PA, neither in model 1 with each explanatory variable nor in model 2 with simultaneous explanatory variables. Existing guidelines for sedentary behavior prevention in children and adolescents target overall sedentary behavior and recreational screen time, with sedentary behavior being a concern as a factor that inhibits PA. Sedentary behaviors are characterized by an energy expenditure of fewer than 1.5 METs in a sitting, reclining, or lying position [55] and can be screen-based (e.g., TV, computer, smartphone, and video games) or non-screen-based (e.g., reading books, doing paper-based homework, and playing board games). These behaviors per se may directly impact metabolic outcomes, but other effects may vary depending on the activity performed while sedentary [12]. Screen-based sedentary behaviors often show adverse associations with a variety of health outcomes, including body composition, cardiometabolic risk, behavioral symptoms, physical fitness, self-esteem, and sleep among school-aged children and youth aged 5–18 years [56–58].

Maintaining and promoting PA among children is an urgent issue for children’s health habits. However, screen-based devices may also provide opportunities for new educational approaches, child engagement, and increased access to education for some students, especially as observed during the coronavirus disease epidemic. Passive parental involvement that limits sedentary activities such as television and video games, may not be effective in school-aged children, and a proactive approach may be required.

### Limitations

Although this study contributes to identifying the association between parental and children’s PA levels, it has several limitations. Since it was a cross-sectional study, causal relationships could not be verified, and further longitudinal validation is required. As we could only identify indicators of limited involvement of caregivers (logistic support, modeling, use of community resources, and restricting access to sedentary activities), it is important to explore additional parental involvement and environmental factors that promote PA in future studies. Moreover, owing to the limited geographic region of the study, it is difficult to generalize our results. Further studies conducted in a wider area and in regions with diverse cultural backgrounds are needed.

## Conclusions

Direct parental logistic support for PA, such as enrollment in specific sports clubs during the school-age period, may encourage children to engage in MVPA, and recommending the use of community resources may encourage them to engage in moderate and light PA, such as walking. In contrast, indirect and negative involvements such as modeling and limiting sedentary activities were not associated with children’s actual PA. Future studies should be performed to investigate further the specific factors that promote PA in children.

## Electronic supplementary material

Below is the link to the electronic supplementary material.

Supplementary Material 1

Supplementary Material 2

## Data Availability

The datasets generated and/or analyzed during the present study are available from the corresponding author upon reasonable request.

## List of Abbreviations

ACTS-MG: Activity support scale for multiple groups
IPAQ: International physical activity questionnaire
IPAQ-SF: International physical activity questionnaire-short form
MET: Metabolic equivalent of task
MVPA: Moderate-to-vigorous physical activity
PA: Physical activity
VIF: Variance inflation factors
WHO: World Health Organization
