# Supplementary material for: Parental support for physical activity and children’s physical activities: a cross-sectional study
Source: BMC Sports Sci Med Rehabil. 2023 Jul 25;15:90. doi: 10.1186/s13102-023-00700-9 (PMC10367251; doi:10.1186/s13102-023-00700-9)
Supplement: Supplementary file 1 — Supplementary Material 1 [file 13102_2023_700_MOESM1_ESM.docx]

**Table S-1. The Activity Support Scale for Multiple Groups (ACTS-MG)**

| Subscales |  |
| --- | --- |
| Logistic Support | I enroll my child in sports teams and clubs such as those of soccer, basketball, and dance. |
|  | I take my child to places where he/she can be active. |
|  | I watch my child play sports or participate in other activities such as martial arts or dance. |
| Modeling | I encourage my child to be physically active by leading by example (by role modeling). |
|  | I exercise or am physically active on a regular basis. |
|  | I enjoy exercise and physical activity. |
| Use of community resources | I encourage my child to use resources in our neighborhood to be active (such as the park and the school). |
|  | I enroll my child in community-based programs (such as Girls and Boys Club, YMCA) where he/she can be active. |
|  | I find ways for my child to be active when school is out by, for example, enrolling him/her in summer camp and after school programs. |
| Restricting access to sedentary activities | I limit how long my child plays video games (including playstation, Xbox, and gameboys). |
|  | I limit how long my child can watch TV or DVDs each day (including educational and non-educational programs). |
|  | I limit how long my child can use the computer for things other than homework (such as playing computer games and surfing the internet). |
