# Supplementary material for: Parental support for physical activity and children’s physical activities: a cross-sectional study
Source: BMC Sports Sci Med Rehabil. 2023 Jul 25;15:90. doi: 10.1186/s13102-023-00700-9 (PMC10367251; doi:10.1186/s13102-023-00700-9)
Supplement: Supplementary file 2 — Supplementary Material 2 [file 13102_2023_700_MOESM2_ESM.docx]

**Table S-2. Association between parental attitudes that support physical activity and children’s physical activities for males (n = 366)**

|  | **Model 1** | | | | | **Model 2** | | | | |
| --- | --- | --- | --- | --- | --- | --- | --- | --- | --- | --- |
|  | **B** | **SE** | **β** | **p** | **Adjusted R^2^** | **B** | **SE** | **β** | **p** | **Adjusted R^2^** |
| **Moderate-to-vigorous physical activity** |  |  |  |  |  |  |  |  |  |  |
| Logistic support | 312.668 | 58.929 | 0.276 | <0.001* | 0.090 | 277.803 | 72.749 | 0.243 | <0.001* | 0.112 |
| Modeling | 82.597 | 40.639 | 0.109 | 0.043* | 0.027 | -40.342 | 47.266 | -0.053 | 0.394 |  |
| Use of community resources | 236.394 | 63.268 | 0.201 | <0.001* | 0.052 | 124.464 | 77.712 | 0.106 | 0.110 |  |
| Limiting sedentary activities | 77.291 | 51.407 | 0.081 | 0.134 | 0.020 | 10.430 | 52.578 | 0.011 | 0.843 |  |
| **Vigorous physical activity** |  |  |  |  |  |  |  |  |  |  |
| Logistic support | 266.428 | 53.619 | 0.261 | <0.001* | 0.073 | 236.389 | 66.331 | 0.229 | <0.001* | 0.093 |
| Modeling | 83.871 | 36.870 | 0.123 | 0.024* | 0.021 | -11.620 | 43.096 | -0.017 | 0.788 |  |
| Use of community resources | 190.368 | 57.745 | 0.179 | 0.001* | 0.036 | 83.062 | 70.856 | 0.079 | 0.242 |  |
| Limiting sedentary activities | 57.080 | 46.720 | 0.066 | 0.223 | 0.010 | -2.395 | 47.939 | -0.003 | 0.960 |  |
| **Moderate physical activity** |  |  |  |  |  |  |  |  |  |  |
| Logistic support | 46.239 | 19.080 | 0.130 | 0.016* | 0.028 | 41.414 | 23.426 | 0.116 | 0.078 | 0.060 |
| Modeling | -1.274 | 12.750 | -0.005 | 0.920 | 0.013 | -28.722 | 15.220 | -0.121 | 0.060 |  |
| Use of community resources | 46.027 | 19.981 | 0.126 | 0.022* | 0.027 | 41.402 | 25.024 | 0.113 | 0.099 |  |
| Limiting sedentary activities | 20.211 | 16.013 | 0.068 | 0.208 | 0.017 | 12.825 | 16.931 | 0.043 | 0.449 |  |
| **Walking** |  |  |  |  |  |  |  |  |  |  |
| Logistic support | 59.711 | 29.519 | 0.110 | 0.044* | 0.010 | 44.742 | 36.466 | 0.081 | 0.221 | 0.035 |
| Modeling | 14.529 | 19.865 | 0.040 | 0.465 | -0.002 | -17.511 | 23.692 | -0.048 | 0.460 |  |
| Use of community resources | 59.655 | 31.211 | 0.105 | 0.057 | 0.007 | 40.647 | 38.954 | 0.072 | 0.298 |  |
| Limiting sedentary activities | 42.525 | 24.951 | 0.093 | 0.089 | 0.004 | 31.105 | 26.355 | 0.068 | 0.239 |  |

Note: Model 1: independent variables were entered individually; Model 2: all independent variables and adjusted variables (children’s sex, family composition, family income, and parental educational attainment) were entered simultaneously.

Abbreviations: unstandardized coefficient (B), standard error (SE), standardized coefficient (β). * Indicates a statistically significant difference.

**Table S-3. Association between parental attitudes that support physical activity and children’s physical activities for females (n = 351)**

|  | **Model 1** | | | | | **Model 2** | | | | |
| --- | --- | --- | --- | --- | --- | --- | --- | --- | --- | --- |
|  | **B** | **SE** | **β** | **p** | **Adjusted R^2^** | **B** | **SE** | **β** | **p** | **Adjusted R^2^** |
| **Moderate-to-vigorous physical activity** |  |  |  |  |  |  |  |  |  |  |
| Logistic support | 143.064 | 38.000 | 0.205 | <0.001* | 0.037 | 161.902 | 50.560 | 0.230 | 0.002* | 0.059 |
| Modeling | 60.215 | 27.986 | 0.119 | 0.032* | 0.009 | 16.885 | 35.480 | 0.033 | 0.634 |  |
| Use of community resources | 37.808 | 40.541 | 0.052 | 0.352 | -0.003 | -50.983 | 50.093 | -0.070 | 0.310 |  |
| Limiting sedentary activities | 3.775 | 35.126 | 0.006 | 0.914 | -0.005 | -30.328 | 38.148 | -0.047 | 0.427 |  |
| **Vigorous physical activity** |  |  |  |  |  |  |  |  |  |  |
| Logistic support | 131.532 | 33.803 | 0.211 | <0.001* | 0.045 | 164.136 | 44.876 | 0.261 | <0.001* | 0.072 |
| Modeling | 46.920 | 24.946 | 0.104 | 0.061 | 0.013 | 7.463 | 31.492 | 0.016 | 0.813 |  |
| Use of community resources | 18.421 | 36.155 | 0.028 | 0.611 | 0.003 | -73.234 | 44.462 | -0.112 | 0.101 |  |
| Limiting sedentary activities | 14.110 | 31.284 | 0.025 | 0.652 | 0.003 | -12.226 | 33.860 | -0.021 | 0.718 |  |
| **Moderate physical activity** |  |  |  |  |  |  |  |  |  |  |
| Logistic support | 11.532 | 13.005 | 0.049 | 0.376 | -0.009 | -2.234 | 17.221 | -0.010 | 0.897 | 0.022 |
| Modeling | 13.295 | 9.567 | 0.078 | 0.166 | -0.003 | 9.422 | 12.085 | 0.055 | 0.436 |  |
| Use of community resources | 19.387 | 13.815 | 0.079 | 0.161 | -0.005 | 22.251 | 17.062 | 0.091 | 0.193 |  |
| Limiting sedentary activities | -10.334 | 11.966 | -0.049 | 0.388 | -0.009 | -18.102 | 12.994 | -0.084 | 0.165 |  |
| **Walking** |  |  |  |  |  |  |  |  |  |  |
| Logistic support | -3.332 | 25.891 | -0.007 | 0.898 | 0.023 | -55.032 | 33.733 | -0.116 | 0.104 | 0.084 |
| Modeling | 22.366 | 18.731 | 0.066 | 0.233 | 0.028 | 21.685 | 23.672 | 0.063 | 0.360 |  |
| Use of community resources | 66.179 | 26.865 | 0.135 | 0.014* | 0.040 | 98.077 | 33.421 | 0.199 | 0.004* |  |
| Limiting sedentary activities | -31.627 | 23.330 | -0.075 | 0.176 | 0.030 | -53.120 | 25.452 | -0.121 | 0.038* |  |

Note: Model 1: independent variables were entered individually; Model 2: all independent variables and adjusted variables (children’s sex, family composition, family income, and parental educational attainment) were entered simultaneously.

Abbreviations: unstandardized coefficient (B), standard error (SE), standardized coefficient (β). * Indicates a statistically significant difference.
